# Supplementary figures and images for: Integrative species delimitation in the common ophiuroid Ophiothrix angulata (Echinodermata: Ophiuroidea): insights from COI, ITS2, arm coloration, and geometric morphometrics
Source: PeerJ. 2023 Jul 17;11:e15655. doi: 10.7717/peerj.15655 (PMC10358340; doi:10.7717/peerj.15655)

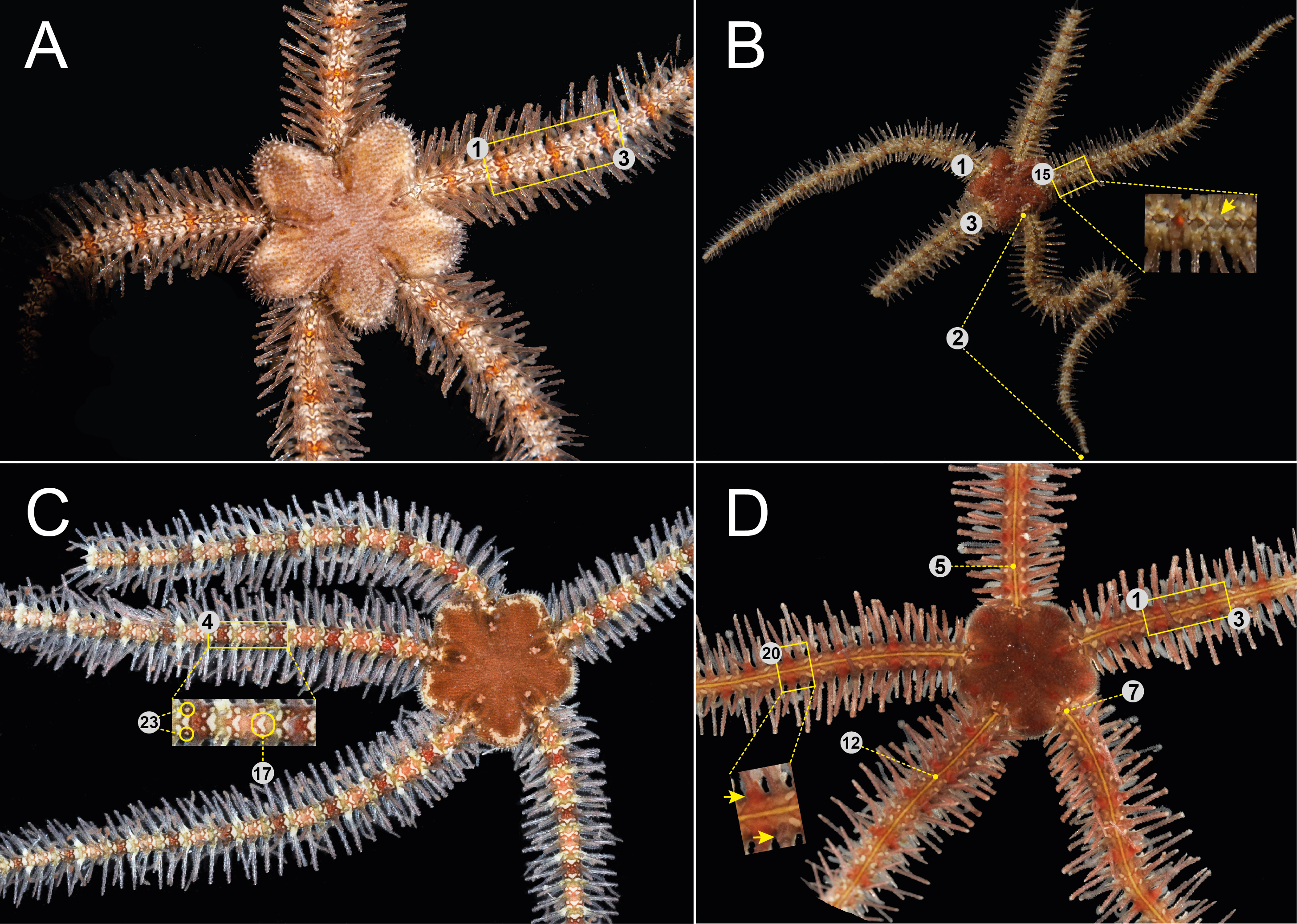

Supplement: Supplemental Information 1 — In vivo, dorsal arm color patterns of 25 characters were used in the parsimony analysis. Characters (Appendix S1): 1, 2, 3, 4, 5, 7, 12, 15, 17, 20, and 23. Outgroup characters are available in Fig. 6. (A) UF7631. (B) UF13163. (C) UF10250. (D) UF10825. Photo credit: Invertebrate Zoology Collection, Florida Museum of Natural History, University of Florida. [file peerj-11-15655-s001.jpg]

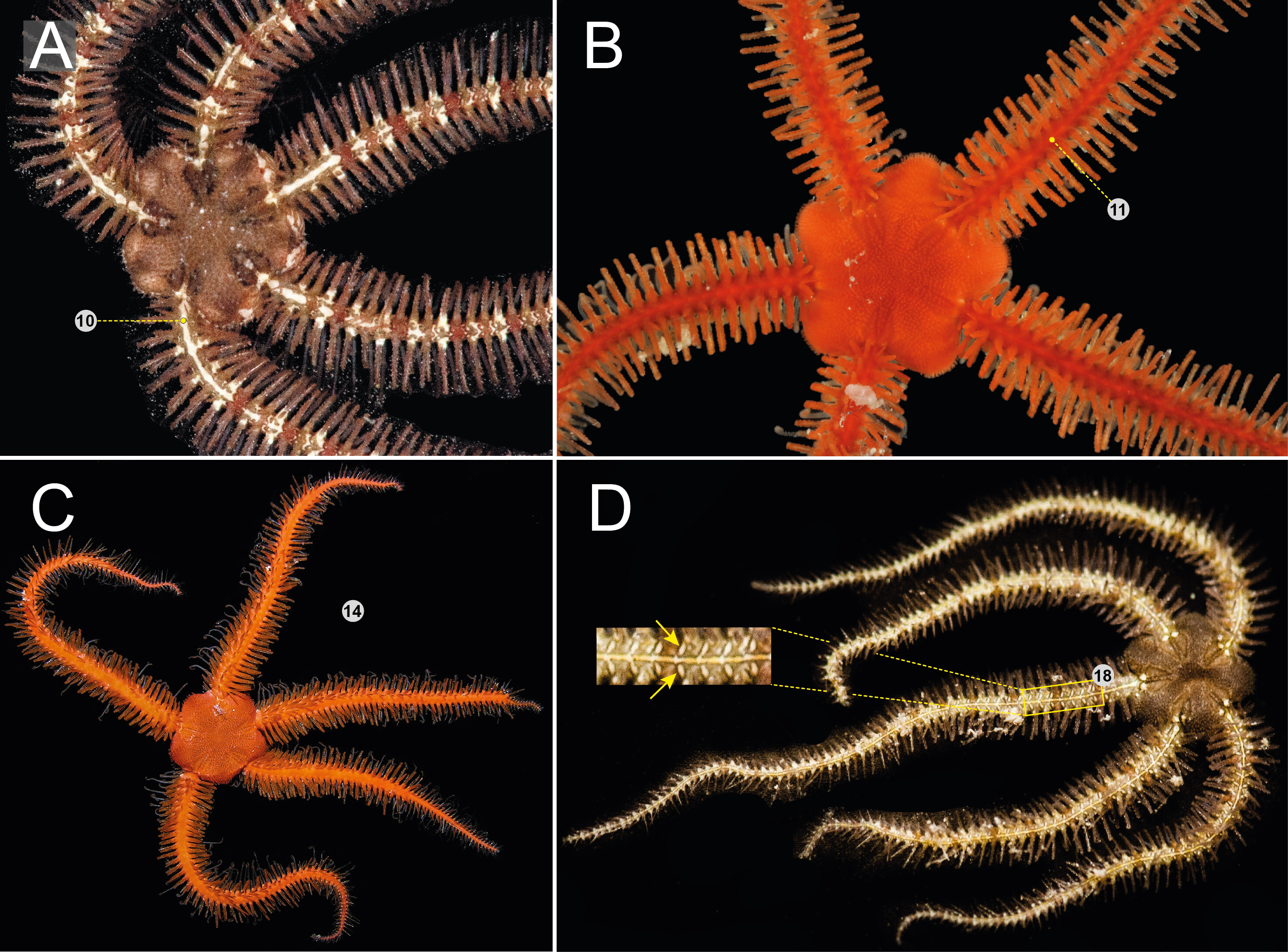

Supplement: Supplemental Information 2 — In vivo, dorsal arm color patterns of 25 characters were used in the parsimony analysis. Characters (Appendix S1): 10, 11, 14, and 18. Outgroup characters are available in Fig. 6. (A) COREPY25a. (B) UF10823. (C) UF9013. (D) COREPY207. Photo credit: Invertebrate Zoology Collection, Florida Museum of Natural History, University of Florida, and Y. Quetzalli Hernández-Díaz. [file peerj-11-15655-s002.jpg]

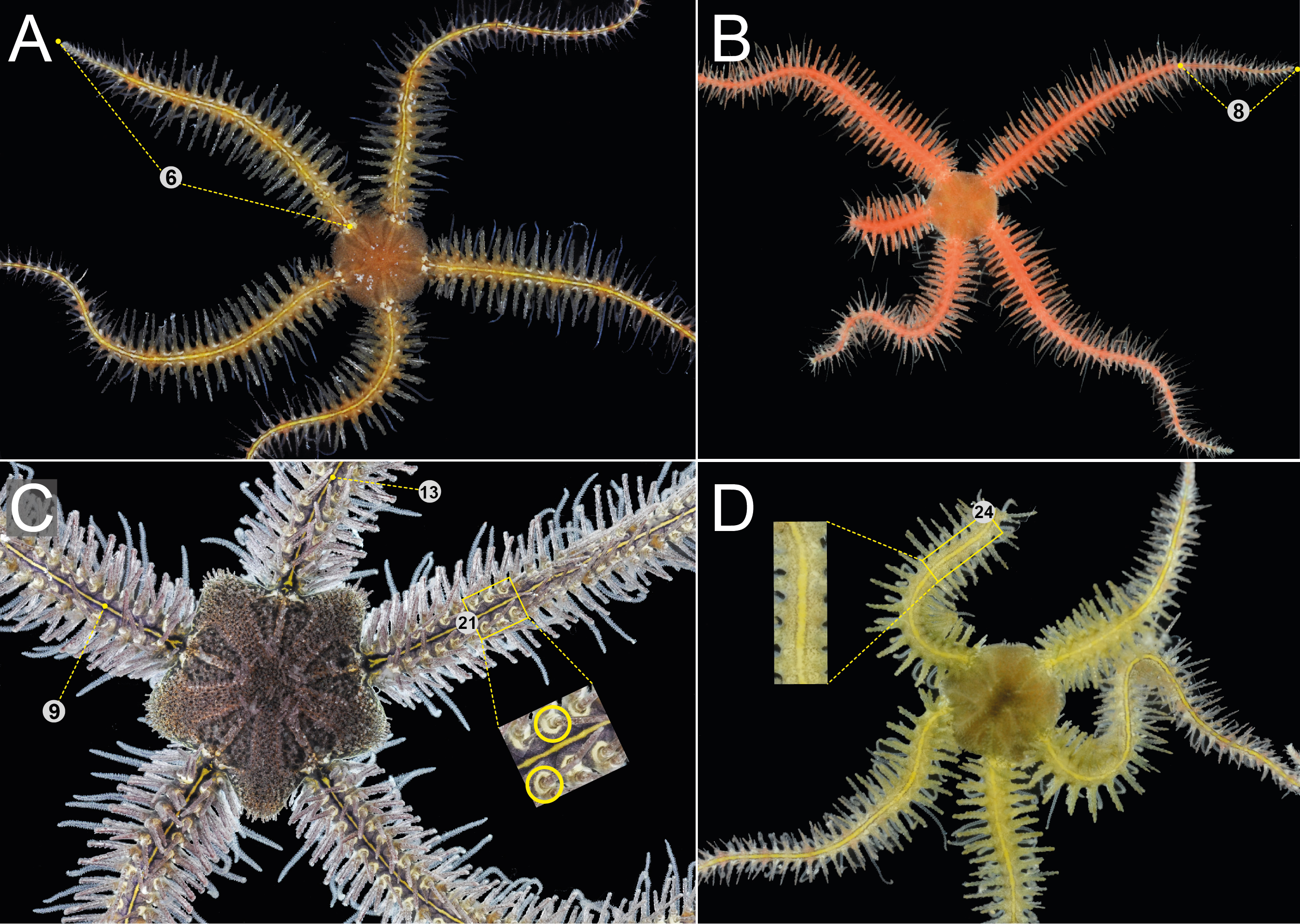

Supplement: Supplemental Information 3 — In vivo, dorsal arm color patterns of 25 characters were used in the parsimony analysis. Characters (Appendix S1): 6, 8, 9, 13, 21, and 24. Outgroup characters are available in Fig. 6. (A) UF11605. (B) UF11953. (C) UF10248. (D) UF13948. Photo credit: Invertebrate Zoology Collection, Florida Museum of Natural History, University of Florida. [file peerj-11-15655-s003.jpg]

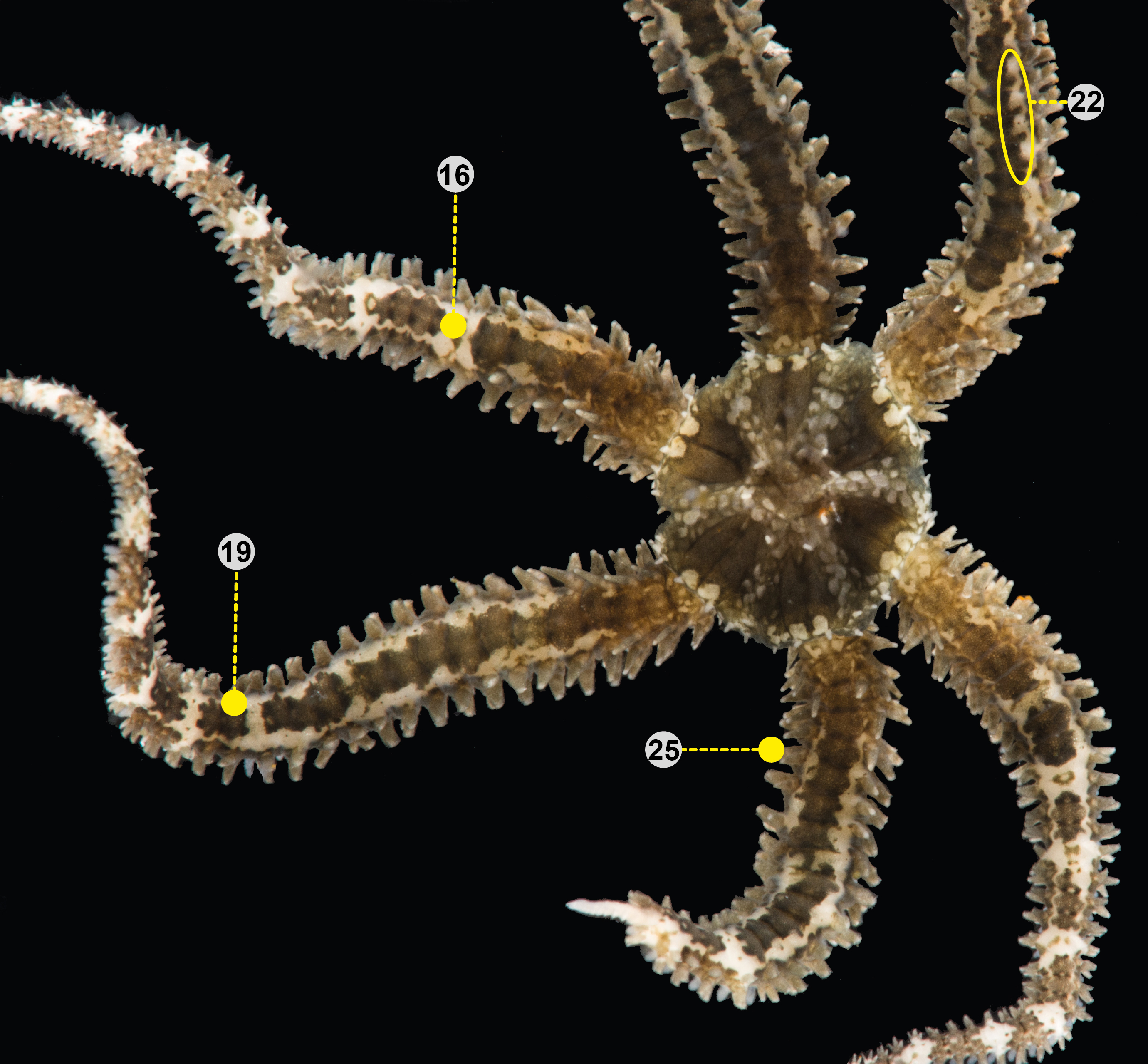

Supplement: Supplemental Information 4 — In vivo, dorsal arm color patterns of 25 characters were used in the parsimony analysis. Ophiactis savignyi ICML-UNAM 18084 used as outgroup in the maximum parsimony cladogram. Characters (Appendix S1): 16, 19, 22, and 25. Photo credit: Y. Quetzalli Hernández-Díaz. [file peerj-11-15655-s004.jpg]
