# Supplementary material for: Integrative species delimitation in the common ophiuroid Ophiothrix angulata (Echinodermata: Ophiuroidea): insights from COI, ITS2, arm coloration, and geometric morphometrics
Source: PeerJ. 2023 Jul 17;11:e15655. doi: 10.7717/peerj.15655 (PMC10358340; doi:10.7717/peerj.15655)
Supplement: Supplemental Information 15 — List of characters of dorsal arm coloration. [file peerj-11-15655-s015.docx]

**S1 Appendix: List of characters of dorsal arm coloration**

**Dorsal Arm**

1. A transversal banding pattern is present: no (0), yes (1).
2. The color bands are visible on all segments of the arm: no (0), yes (1), without bands (2).
3. Banding pattern with two colors, repeats every four segments: no (0), yes (1), without bands (2).
4. Banding pattern with three colors, repeats every four segments: no (0), yes (1), without bands (2).
5. Longitudinal Line (LL) is present: no (0), yes (1).
6. Longitudinal Line is visible on all segments of the arm: no (0), yes (1), without LL (2).
7. Longitudinal Line starts on the first dorsal segment: no (0), yes (1), without LL (2).
8. Longitudinal Line starts at mid-arm or in distal segments: no (0), yes (1), without LL (2).
9. Longitudinal Line is yellow: no (0), yes (1), without LL (2).
10. Longitudinal Line is white: no (0), yes (1), without LL (2).
11. Longitudinal Line has the same color as the segment but lighter: no (0), yes (1), without LL (2).
12. Longitudinal Line is bordered by parallel lines: no (0), yes (1), without LL (2).
13. Parallel lines bordering longitudinal lines are dark: no (0), yes (1), without LL (2).

**Dorsal Arms Plates**

1. Base color is uniform on all arms: no (0), yes (1).
2. Rhomboidal stain is present: no (0), yes (1).
3. A white stain can cover a large plate area: no (0), yes (1).
4. A triangular white stain in the middle of the plate is present: no (0), yes (1).
5. White spots or stains bordering the distal edge: no (0), yes (1).
6. White and green patron mix is present: no (0), yes (1).

**Arm Spine Base and Lateral Arm Plate**

1. Arm Spine Base color is the same as the Dorsal Arm Plate: no (0), yes (1).
2. Arm Spine Base color is white o lighter: no (0), yes (1).
3. Arm Spine Base color presents a white and green mix: no (0), yes (1).
4. Lateral Arm Plate presents white circular spots at the dorsal proximal edge (at least in some segments): no (0), yes (1).
5. Lateral Arm Plate presents dark mini dots: no (0), yes (1).
6. Lateral Arm Plate presents a white and green mix: no (0), yes (1).
