# Supplementary material for: Integrative species delimitation in the common ophiuroid Ophiothrix angulata (Echinodermata: Ophiuroidea): insights from COI, ITS2, arm coloration, and geometric morphometrics
Source: PeerJ. 2023 Jul 17;11:e15655. doi: 10.7717/peerj.15655 (PMC10358340; doi:10.7717/peerj.15655)
Supplement: Supplemental Information 16 — Character matrix of the 47 individuals and 25 characters used in the Parsimony analysis. Out = outgroup. [file peerj-11-15655-s016.docx]

**S2 Appendix:** Character matrix of the 47 individuals and 25 characters used in the Parsimony analysis. Out = outgroup.

| **No.** | **Specimens** | **Clade** | **Characters** | | | | | | | | | | | | | | | | | | | | | | | | | | |
| --- | --- | --- | --- | --- | --- | --- | --- | --- | --- | --- | --- | --- | --- | --- | --- | --- | --- | --- | --- | --- | --- | --- | --- | --- | --- | --- | --- | --- | --- |
|  |  |  | **1** | **2** | **3** | **4** | **5** | **6** | **7** | **8** | **9** | **0** |  | **1** | **2** | **3** | **4** | **5** | **6** | **7** | **8** | **9** | **0** |  | **1** | **2** | **3** | **4** | **5** |
| 1 | *O. savignyi* | Out | 1 | 0 | 0 | 0 | 0 | 2 | 2 | 2 | 2 | 2 |  | 2 | 2 | 2 | 0 | 0 | 1 | 0 | 0 | 1 | 0 |  | 0 | 1 | 0 | 0 | 1 |
| 2 | UF7631 | 1A | 1 | 1 | 1 | 0 | 0 | 2 | 2 | 2 | 2 | 2 |  | 2 | 2 | 2 | 0 | 1 | 0 | 0 | 0 | 0 | 1 |  | 0 | 0 | 0 | 0 | 0 |
| 3 | UF7632 | 1A | 1 | 1 | 1 | 0 | 0 | 2 | 2 | 2 | 2 | 2 |  | 2 | 2 | 2 | 0 | 1 | 0 | 0 | 0 | 0 | 1 |  | 0 | 0 | 0 | 0 | 0 |
| 4 | UF7633 | 1A | 1 | 1 | 1 | 0 | 0 | 2 | 2 | 2 | 2 | 2 |  | 2 | 2 | 2 | 0 | 1 | 0 | 0 | 0 | 0 | 1 |  | 0 | 0 | 0 | 0 | 0 |
| 5 | UF8961 | 1A | 1 | 1 | 1 | 0 | 1 | 1 | 1 | 0 | 1 | 0 |  | 0 | 1 | 1 | 0 | 0 | 0 | 0 | 1 | 0 | 1 |  | 0 | 0 | 0 | 0 | 0 |
| 6 | UF8971 | 1A | 1 | 0 | 1 | 0 | 1 | 1 | 1 | 0 | 1 | 0 |  | 0 | 1 | 1 | 1 | 0 | 0 | 0 | 0 | 0 | 1 |  | 0 | 0 | 1 | 0 | 0 |
| 7 | UF8973 | 1A | 1 | 0 | 1 | 0 | 1 | 1 | 1 | 0 | 1 | 0 |  | 0 | 1 | 1 | 1 | 0 | 0 | 0 | 1 | 0 | 1 |  | 0 | 0 | 0 | 0 | 0 |
| 8 | UF10247 | 1A | 1 | 0 | 1 | 0 | 1 | 1 | 1 | 0 | 1 | 0 |  | 0 | 1 | 1 | 0 | 0 | 0 | 0 | 0 | 0 | 0 |  | 1 | 0 | 0 | 1 | 0 |
| 9 | UF10779 | 1A | 1 | 1 | 1 | 0 | 1 | 1 | 1 | 0 | 1 | 0 |  | 0 | 1 | 1 | 0 | 0 | 0 | 0 | 0 | 0 | 1 |  | 0 | 0 | 0 | 0 | 0 |
| 10 | UF10823 | 1A | 0 | 2 | 2 | 2 | 1 | 1 | 0 | 0 | 0 | 0 |  | 1 | 0 | 2 | 1 | 0 | 0 | 0 | 0 | 0 | 1 |  | 0 | 0 | 0 | 0 | 0 |
| 11 | UF10825 | 1A | 1 | 1 | 1 | 0 | 1 | 1 | 1 | 0 | 1 | 0 |  | 0 | 1 | 1 | 0 | 0 | 0 | 0 | 1 | 0 | 1 |  | 0 | 0 | 1 | 0 | 0 |
| 12 | UF11584 | 1A | 1 | 0 | 1 | 0 | 1 | 1 | 1 | 0 | 1 | 0 |  | 0 | 1 | 1 | 0 | 0 | 0 | 0 | 0 | 0 | 1 |  | 0 | 0 | 1 | 0 | 0 |
| 13 | UF11605 | 1A | 1 | 0 | 1 | 0 | 1 | 1 | 1 | 0 | 1 | 0 |  | 0 | 1 | 1 | 0 | 0 | 0 | 0 | 0 | 0 | 1 |  | 0 | 0 | 1 | 0 | 0 |
| 14 | UF11606 | 1A | 1 | 1 | 1 | 0 | 1 | 1 | 1 | 0 | 1 | 0 |  | 0 | 1 | 1 | 0 | 0 | 0 | 0 | 0 | 0 | 1 |  | 0 | 0 | 1 | 0 | 0 |
| 15 | UF11613 | 1A | 1 | 1 | 1 | 0 | 1 | 1 | 1 | 0 | 1 | 0 |  | 0 | 1 | 1 | 0 | 0 | 0 | 0 | 0 | 0 | 1 |  | 0 | 0 | 1 | 0 | 0 |
| 16 | UF11621 | 1A | 0 | 2 | 2 | 2 | 1 | 1 | 1 | 0 | 1 | 0 |  | 0 | 1 | 1 | 0 | 0 | 0 | 0 | 1 | 0 | 1 |  | 0 | 0 | 1 | 1 | 0 |
| 17 | UF11637 | 1A | 0 | 2 | 2 | 2 | 1 | 0 | 0 | 1 | 1 | 0 |  | 0 | 1 | 1 | 1 | 0 | 0 | 0 | 0 | 0 | 1 |  | 0 | 0 | 0 | 0 | 0 |
| 18 | UF11953 | 1A | 1 | 0 | 1 | 0 | 1 | 0 | 0 | 1 | 1 | 0 |  | 0 | 1 | 1 | 1 | 0 | 0 | 0 | 0 | 0 | 1 |  | 0 | 0 | 0 | 1 | 0 |
| 19 | UF13161 | 1A | 0 | 2 | 2 | 2 | 0 | 2 | 2 | 2 | 2 | 2 |  | 2 | 2 | 2 | 1 | 0 | 0 | 0 | 0 | 0 | 1 |  | 0 | 0 | 0 | 0 | 0 |
| 20 | UF13163 | 1A | 1 | 1 | 1 | 0 | 0 | 2 | 2 | 2 | 2 | 2 |  | 2 | 2 | 2 | 0 | 1 | 0 | 0 | 0 | 0 | 1 |  | 0 | 0 | 0 | 0 | 0 |
| 21 | UF13948 | 1A | 1 | 0 | 1 | 0 | 1 | 1 | 1 | 0 | 1 | 0 |  | 0 | 1 | 1 | 0 | 0 | 0 | 0 | 0 | 0 | 1 |  | 0 | 0 | 0 | 1 | 0 |
| 22 | UF7634 | 1B | 1 | 1 | 1 | 0 | 1 | 1 | 1 | 0 | 1 | 0 |  | 0 | 1 | 1 | 0 | 0 | 0 | 0 | 0 | 0 | 1 |  | 0 | 0 | 0 | 1 | 0 |
| 23 | UF8963 | 1B | 1 | 1 | 1 | 0 | 1 | 1 | 1 | 0 | 1 | 0 |  | 0 | 1 | 1 | 0 | 0 | 0 | 0 | 0 | 0 | 1 |  | 0 | 0 | 1 | 0 | 0 |
| 24 | UF8972 | 1B | 1 | 0 | 1 | 0 | 1 | 1 | 1 | 0 | 1 | 0 |  | 0 | 1 | 1 | 0 | 0 | 0 | 0 | 0 | 0 | 0 |  | 1 | 0 | 0 | 0 | 0 |
| 25 | UF9013 | 1B | 0 | 2 | 2 | 2 | 0 | 2 | 2 | 2 | 2 | 2 |  | 2 | 2 | 2 | 1 | 0 | 0 | 0 | 0 | 0 | 1 |  | 0 | 0 | 0 | 0 | 0 |
| 26 | UF10822 | 1B | 1 | 1 | 1 | 0 | 1 | 1 | 1 | 0 | 1 | 0 |  | 0 | 1 | 1 | 0 | 0 | 0 | 0 | 0 | 0 | 1 |  | 0 | 0 | 1 | 0 | 0 |
| 27 | UF10829 | 1B | 1 | 1 | 1 | 0 | 1 | 1 | 1 | 0 | 1 | 0 |  | 0 | 1 | 1 | 0 | 0 | 0 | 0 | 1 | 0 | 1 |  | 0 | 0 | 0 | 0 | 0 |
| 28 | UF11592 | 1B | 1 | 0 | 1 | 0 | 1 | 0 | 0 | 1 | 1 | 0 |  | 0 | 1 | 1 | 1 | 0 | 0 | 0 | 0 | 0 | 1 |  | 0 | 0 | 0 | 0 | 0 |
| 29 | UF11593 | 1B | 1 | 1 | 1 | 0 | 1 | 1 | 1 | 0 | 1 | 0 |  | 0 | 1 | 1 | 0 | 0 | 0 | 0 | 1 | 0 | 1 |  | 0 | 0 | 1 | 1 | 0 |
| 30 | UF11608 | 1B | 1 | 1 | 1 | 0 | 1 | 1 | 1 | 0 | 1 | 0 |  | 0 | 1 | 1 | 0 | 0 | 0 | 0 | 0 | 0 | 1 |  | 0 | 0 | 1 | 0 | 0 |
| 31 | UF11649 | 1B | 1 | 0 | 1 | 0 | 1 | 1 | 1 | 0 | 0 | 0 |  | 1 | 0 | 2 | 1 | 0 | 0 | 0 | 0 | 0 | 1 |  | 0 | 0 | 0 | 1 | 0 |
| 32 | UF17827 | 1B | 1 | 1 | 1 | 0 | 1 | 1 | 1 | 0 | 1 | 0 |  | 0 | 1 | 1 | 0 | 0 | 0 | 0 | 0 | 0 | 0 |  | 1 | 0 | 0 | 0 | 0 |
| 33 | UF8974 | 2A | 0 | 2 | 2 | 2 | 1 | 1 | 1 | 0 | 0 | 0 |  | 1 | 1 | 1 | 1 | 0 | 0 | 0 | 0 | 0 | 1 |  | 0 | 0 | 0 | 0 | 0 |
| 34 | UF9010 | 2A | 0 | 2 | 2 | 2 | 1 | 0 | 0 | 1 | 1 | 0 |  | 0 | 1 | 1 | 1 | 0 | 0 | 0 | 0 | 0 | 1 |  | 0 | 0 | 0 | 0 | 0 |
| 35 | UF9017 | 2A | 0 | 2 | 2 | 2 | 1 | 1 | 1 | 0 | 1 | 0 |  | 0 | 1 | 1 | 0 | 0 | 0 | 0 | 0 | 0 | 0 |  | 1 | 0 | 0 | 0 | 0 |
| 36 | UF10248 | 2A | 1 | 0 | 1 | 0 | 1 | 1 | 1 | 0 | 1 | 0 |  | 0 | 1 | 1 | 0 | 0 | 0 | 0 | 0 | 0 | 0 |  | 1 | 0 | 0 | 1 | 0 |
| 37 | UF10276 | 2A | 1 | 0 | 1 | 0 | 1 | 1 | 1 | 0 | 1 | 0 |  | 0 | 1 | 1 | 0 | 0 | 0 | 0 | 0 | 0 | 0 |  | 1 | 0 | 0 | 0 | 0 |
| 38 | UF19463 | 2A | 0 | 2 | 2 | 2 | 1 | 1 | 1 | 0 | 1 | 0 |  | 0 | 1 | 1 | 1 | 0 | 0 | 0 | 0 | 0 | 1 |  | 0 | 0 | 0 | 0 | 0 |
| 39 | COREPY07 | 2A | 1 | 1 | 1 | 0 | 1 | 1 | 1 | 0 | 1 | 0 |  | 0 | 1 | 1 | 0 | 0 | 0 | 0 | 1 | 0 | 1 |  | 0 | 0 | 0 | 0 | 0 |
| 40 | COREPY025a | 3 | 1 | 1 | 0 | 1 | 1 | 0 | 1 | 0 | 0 | 1 |  | 0 | 0 | 2 | 0 | 0 | 0 | 1 | 1 | 0 | 1 |  | 0 | 0 | 0 | 0 | 0 |
| 41 | COREPY081a | 3 | 1 | 0 | 0 | 1 | 0 | 2 | 2 | 2 | 2 | 2 |  | 2 | 2 | 2 | 0 | 0 | 0 | 1 | 1 | 0 | 1 |  | 0 | 0 | 1 | 1 | 0 |
| 42 | COREPY081b | 3 | 1 | 0 | 0 | 1 | 0 | 2 | 2 | 2 | 2 | 2 |  | 2 | 2 | 2 | 1 | 0 | 0 | 1 | 1 | 0 | 1 |  | 0 | 0 | 0 | 0 | 0 |
| 43 | COREPY714 | 3 | 1 | 1 | 0 | 1 | 0 | 2 | 2 | 2 | 2 | 2 |  | 2 | 2 | 2 | 0 | 0 | 0 | 1 | 1 | 0 | 1 |  | 0 | 0 | 1 | 0 | 0 |
| 44 | COREPY717 | 3 | 1 | 1 | 0 | 1 | 0 | 2 | 2 | 2 | 2 | 2 |  | 2 | 2 | 2 | 0 | 0 | 0 | 1 | 1 | 0 | 1 |  | 0 | 0 | 1 | 0 | 0 |
| 45 | UF10250 | 3 | 1 | 1 | 0 | 1 | 0 | 2 | 2 | 2 | 2 | 2 |  | 2 | 2 | 2 | 0 | 0 | 0 | 1 | 1 | 0 | 1 |  | 0 | 0 | 1 | 0 | 0 |
| 46 | UF16896 | 3 | 1 | 1 | 1 | 0 | 0 | 2 | 2 | 2 | 2 | 2 |  | 2 | 2 | 2 | 0 | 0 | 0 | 0 | 1 | 0 | 1 |  | 0 | 0 | 1 | 0 | 0 |
| 47 | UF16993 | 3 | 1 | 0 | 0 | 1 | 0 | 2 | 2 | 2 | 2 | 2 |  | 2 | 2 | 2 | 0 | 0 | 0 | 0 | 1 | 0 | 1 |  | 0 | 0 | 1 | 0 | 0 |
